# Horizontal Stacking of *PAPhy*_a Cisgenes in Barley Is a Potent Strategy for Increasing Mature Grain Phytase Activity

**DOI:** 10.3389/fpls.2020.592139

**Published:** 2020-10-23

**Authors:** Inger Baeksted Holme, Claus Krogh Madsen, Toni Wendt, Henrik Brinch-Pedersen

**Affiliations:** Department of Agroecology, Aarhus University Flakkebjerg, Slagelse, Denmark

**Keywords:** Barley, cisgene, field trial, *HvPAPhy_a*, mature grain phytase activity, horizontal stacking

## Abstract

Mature grain phytase activity (MGPA) in the Triticea tribe cereals has evolved through gene duplications and neo-functionalization of the purple acid phosphatase phytase gene (*PAPhy*) in a common ancestor. Increased gene copy number of the *PAPhy_a* gene expressed during seed development has augmented the MGPA in cereals like rye and wheat. PAPhy_a phytase is highly stable and a potent enzyme in feed. However, barley only contains one *HvPAPhy_a* gene and the MGPA levels needs to be increased to substitute for the addition of microbial phytases to the feed. A substantial increase in MGPA for cisgenic barley was achieved with one extra homozygous *HvPAPhy_a* insert when the plants were grown in the greenhouse. In the current study, the stability of increased MGPA was confirmed in open field grown cisgenic barley. Furthermore, the gene dose response of phytase cisgenes from three different cisgenic barley plants were horizontally stacked. Cisgenic barley with 0, 1, 2, 3, 4, and 6 extra *HvPAPhy_a* inserts demonstrated a perfect positive linear correlation with the level of MGPA. The current study provides new insight into the potential of stacking of cisgenes in crops and suggests cisgene stacking as a versatile strategy for crop improvement.

## Introduction

Phytases (myo-inositol hexakisphosphate 3-,6- and 5-phosphohydrolase, EC 3.1.3.8, EC 3.1.3.26, and EC 3.1.3.72) are phosphatases that can initiate the stepwise hydrolysis of phytate, the main storage form of phosphate in seeds and the major antinutritional factor for bio-availability of micronutrient in food and feed (Lott, 1984). A high phytase activity in food and feedstuffs is desirable to ensure high bioavailability of phytate bound phosphate and counter the antinutritional effects of non-digested phytate. Hydrolysis of phytate provides not only bioavailable phosphate but also prevents phytate-chelation of nutritional important cations from the diet in the digestive tract (Brinch-Pedersen et al., 2002, 2014; Torres et al., 2005; Neal et al., 2013).

The level of pre-formed phytase, constituting the mature grain phytase activity (MGPA), varies considerably between cereal species. Substantial activity is present in mature grains of cereals of the Triticeae tribe such as wheat (*Triticum aestivum* L.), barley (*Hordeum vulgare* L.) and rye (*Secale cereale* L.). Moreover, variation also occurs within species, with up to two fold differences between cultivars of wheat. By contrast, non-Triticeae cereals such as maize (*Zea mays* L.), rice (*Oryza sativa* L.) and oat (*Avena sativa L.*) have very little MGPA (Madsen and Brinch-Pedersen, 2019).

Phytases in cereals has been found among the groups of histidine acid phosphatases (HAPhy) and the purple acid phosphatases (PAPhy) (Dionisio et al., 2007, 2011). However, the main type contributing to MGPA are of the PAPhy type. During evolution of the Triticeae tribe cereals, a single *PAPhy* gene has undergone a lineage specific gene duplication resulting in a set of paralogs *PAPhy_a* and *PAPhy_b* (Madsen et al., 2013, 2017). *PAPhy_a* is located on chromosome 5. It is mainly expressed during grain filling and CRISPR/Cas9 induced knock-out of the *PAPhy_a* in barley uncovered that it accounts for almost all MGPA (Madsen et al., 2013; Holme et al., 2017b). *PAPhy_b* is located on chromosome 3H and is mainly expressed during seed germination accounting for the *de novo* phytase activity synthesized during germination (Dionisio et al., 2011; Madsen et al., 2013). However, further MGPA increase through gene duplications are found in *Secale* (including cultivated rye with 2 *PAPhy_a* type plus 1 *PAPhy_b* type) and in the polyploid *Triticum* (homeologs, 2 or 3 *PAPhy_a* and 2 or 3 *PAPhy_b* loci in tetra- or hexaploids, respectively). These duplicated genes are still highly conserved and has maintained their function (Madsen et al., 2013). Rye is diploid like barley but because of its two *PAPhy_a* loci it has a substantial higher MGPA than barley (Viveros et al., 2000; Madsen et al., 2013). The evolution of MGPA demonstrates a significant potential of elevating a specific enzyme activity through increasing the number gene copies for the corresponding enzyme.

From the application perspective, the cereal PAPhy_a has a range of interesting properties and potentials. The pH optimum of 5.5 of the PAPhy _a enzyme is well suited for the digestive system of monogastics, particularly the small intestine. This pH optimum is the same as for *Aspergillus ficuum* phytase, a popular microbial feed enzyme. Also, the catalytic efficiency K_m_/K_cat_ of 722 × 10^4^ s^–1^M^–1^ almost reach that of *A. ficuum* phytase (K_m_/K_cat_ of 1290 × 10^4^ s^–1^M^–1^) (Madsen and Brinch-Pedersen, 2019). The potential of PAPhy_a in feed was confirmed in a recent broiler feeding study involving a mutant wheat (HighPhy wheat) with a two to three-fold increase in MGPA reaching 6200 FTU/kg flour. This study showed that the partly replacement of conventional wheat with HighPhy wheat improved calcium and phosphorous digestibility. Moreover, complete replacement with HighPhy wheat significantly outperformed a control to which the standard dose of microbial phytase was added (Brinch-Pedersen et al., 2012; Scholey et al., 2017).

A similar mutant is not available in barley, but increased MGPA can be achieved by introduction of an extra *PAPhy_a* through cisgenesis (Holme et al., 2012). Cisgenesis is a concept defined by Schouten et al. (2006a, b) to describe a restricted application of transformation technology. In contrast to transgenesis where genes and DNA sequences can be moved between any species, the cisgenesis concept is based on the exclusive use of genetic material from the same species or genetic material from closely related species capable of sexual hybridization. Also, the cisgene has to be an unmodified copy of the natural gene and include the native promoter, introns and terminator of the gene, basically a duplication of the native gene of interest. Cisgenic barley holding one extra homozygous insert of the *HvPAPhy_a* endogenous gene, essentially resembles the evolutionary duplication of *PAPhy_a* for higher MGPA as seen in, e.g., rye.

In the current study, we aimed to confirm the performance of cisgenic phytase barley under field conditions, study the gene dose response of stacked phytase cisgenes in barley and uncover the potential of adding more gene copies for a desirable trait. Our results add to the understanding of phytase gene duplication events in cereals and demonstrates a significant potential of cisgenesis as a tool for achieving more efficient crops.

## Materials and Methods

### Field Trials

#### Plant Material for Field Trials

For field trials, grains of the cisgenic plant line PAP07 cv Golden Promise homozygous for an extra copy of the *HvPAPhy_a* gene was used. This plant line was previously generated by *Agrobacterium tumefaciens*-mediated transformation using a cisgenic vector construct. Briefly, a genomic clone of *HvPAPhy_a* was placed in the pCLEAN-G185 vector. In addition to the coding sequence of 2266 bp, the clone included 2000 bp upstream sequence with the promoter and 800 bp downstream sequence with the terminator. The construct was used for *Agrobacterium*-mediated transformation along with the pCLEAN-S166 vector which carried the hygromycin selection gene. By having the selection gene and *HvPAPhy_a* on separate T-DNAs it was possible to obtain marker free plants though segregation in the T_1_-generation. The cisgenic plant PAP07 was characterized and described in detail in Holme et al. (2012). Additionally, non-transformed Golden Promise was used as control.

#### Performance of Field Trials

Grains of the cisgenic PAP07 cv Golden Promise were sown in a 45 m^2^ field plot. The cisgenic field plot was surrounded by a 1.5 m border of non-cisgenic barley to counter edge effects and to fulfill regulatory requirements. A 45 m^2^ field plot of non-cisgenic Golden Promise with a 1.5 m border was also established 150 m away from the cisgenic field plot as a control (Supplementary Figure S1).

**FIGURE 1:**
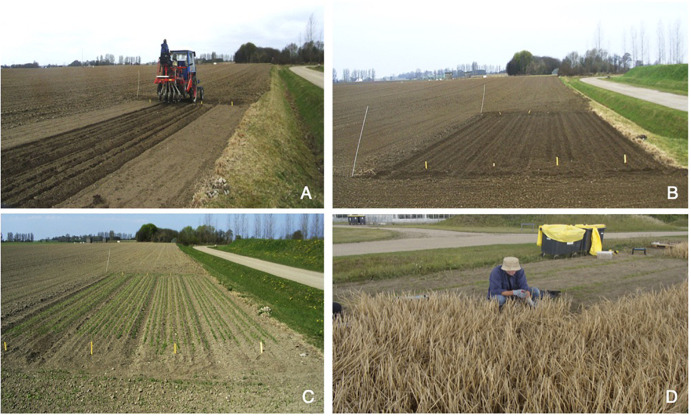
Pictures showing the sowing and harvest of the cisgenic field plot. **(A)** Sowing of the cisgenic plot. **(B)** The cisgenic field plot three days after sowing. **(C)** Germination of seedlings in the cisgenic field plot three weeks after sowing. **(D)** Hand harvest of the cisgenic barley field plot four months after sowing.

Grains were sown at a sowing rate of 16 g/m^2^ to achieve an approximate density of 250 plants/m^2^. Plants were grown to maturity and the spikes of the cisgenic field plot were manually harvested using scissors (Figure 1). In order to get an overview of the variation of MGPA within the field plot, the cisgenic plot was divided into three areas where all spikes and later grains were pooled (Supplementary Figure S1). The collected spikes were threshed in a small scale threshing machine (Wintersteiger Id 180 St4, Wintersteiger AG, Eging). All handling of the cisgenic barley material during sowing, growth, harvest and storage was done according to the requirements of the Directive 2001/18/EC on the deliberate release of genetically modified organisms (GMOs) into the environment and surveilled by the Danish Environmental Protection Agency. For the non-cisgenic control field, all grains were pooled when harvested mechanically.

### Stacking

#### Plant Material for Stacking of HvPAPhy_a Inserts

For gene stacking, three lines (PAP07, PAP05 and PAP03) were used as parents. All lines were generated as described previously (Holme et al., 2012) and each of the lines contains a single *HvPAPhy_a* insert. The flanking left border (LB) sequences were identified for each T-DNA insert in the three lines (Supplementary Figure S2). The LB-flanking regions in the plant lines PAP07 and PAP05 were amplified using the DNA Walking SpeedUpTMPremix Kit (Seegene Germany) as previously described (Holme et al., 2012). The LB flanking region of PAP03 was isolated by Inverted PCR as described by Madsen et al. (2013). For insert mapping, the flanking regions were blasted against the ‘assembly2_WGSBarke’ using the barley IPK Barley Blast Server (IPK-Gartersleben, Germany).

#### Crosses and Detection of the HvPAPhy_a Inserts by PCR of the Flanking Regions

Primers amplifying a DNA fragment starting in the left border flanking region and ending in the *HvPAPhy_a* inserts were designed and used to detect the presence of the *HvPAPhy_a* inserts in the progeny plants after crossing (Supplementary Figure S2; Supplementary Table S1).

All parent plants and progenies of the stacking experiment were grown in the greenhouse. Initially PAP07-plants and PAP05-plants were crossed and nine F_1_-grains from this cross were germinated (Supplementary Table S2). PCR of the LB-flanking sequences were used to check for the presence of both *HvPAPhy_a* inserts in the F_1_-plants (Supplementary Table S1). Two F_1_-plants of the PAP07 and PAP05 cross were then crossed with PAP03. From these crosses eight F_1_-grains were obtained (Supplementary Table S2). These F_1_ seeds were germinated and investigated for the presence of the three *HvPAPhy_a* inserts using PCR of the LB-flanking sequences for each *HvPAPhy_a* insert (Supplementary Table S1).

#### Double Haploid Production by Anther Culture

Anther culture was performed from donor plants showing all three *HvPAP_a* inserts. As the production of doubled haploid lines (DH-lines) through microspore or anther culture from Golden Promise is generally rather low (Coronado et al., 2005), a more successful production was anticipated if more than just only one donor plant was used. Twenty-five seeds from a self-pollinated F_1_-plant containing all three *HvPAPhy_a* inserts were germinated and genotyped by PCR of the LB-flanking sequences for the presence of the three inserts (data not shown). Spikes from seven plants containing all three inserts were then collected and used for anther culture. Spikes chosen randomly from the seven F_2_-plants were harvested and investigated for the developmental stage of the microspores using acetocarmine staining and microscopy. Spikes where most of the microspores were at the uninucleate stage were selected for further culture. The spikes (still within the leaf sheets) were sterilized by spraying with 70% ethanol. A cold pretreatment combined with a starvation pretreatment of the immature pollen was applied as previously described for successfully DH-line production in barley cv. Golden Promise (Coronado et al., 2005). For the cold treatment, the spikes were excised and placed in one room of a 9 cm Petri dish split in two rooms. A drop of water was added to the other room (2 spikes in one Petri dish). The dishes were stored at 4°C in the dark. After 3 weeks, the cold treated anthers were isolated under a microscope and placed on a medium containing 0.7 M mannitol, 40 mM CaCl_2_ and 8 g/l agarose (Sea plaque) (Castillo et al., 2000). The cultures were incubated in the dark at 25°C. After 2 days the anthers were moved to 5 cm Petri dishes containing 5 ml of MS-basal salts without NH_4_NO_3_ (Duchefa M0238), 165 mg/l NH_4_NO_3_, 1 mg/l BAP, 750 mg/l glutamine, 0.4 ml Thiamine, 60 g/l maltose and 200 g/l Ficoll 400 (Olsen, 1987). Twenty anthers from two spikes were placed in each Petri dish and the dishes were subsequently incubated in the dark. Embryos developed within the next 21 to 40 days. These were subsequently transferred to regeneration medium containing MS-basal salts without NH_4_NO_3_, 165 mg/l NH_4_NO_3_, 0.4 mg/l BAP, 750 mg/l glutamine, 0.4 ml Thiamine, 30 g/l maltose and 3 g/l phytagel and incubated at 25°C in the light. Green plantlets approximately 2 cm long were moved to rooting medium as they developed. The rooting medium contained MS-basal salts without NH_4_NO_3_, 165 mg/l NH_4_NO_3_, 146 mg/l glutamine, 30 g/l maltose and 3 g/l phytagel. Rooted green plantlets were transferred to the greenhouse and plants surviving the greenhouse transfer were genotyped one month after the greenhouse transfer (Supplementary Table S2). When the plants were close to maturity, plants without seed setting were discarded (visually scored as either haploids, tetraploids or aneuploids) so that only DH-lines were included in the study.

### Phytase Activity in Mature Grains of the Cisgenic Plants

The mature grain phytase activity (MGPA) was analyzed in the crude protein extract from milled mature grains of parental plants, F_1_-plants and DH-lines according to Brinch-Pedersen et al. (2000) and Engelen et al. (1994). Random samples of 20–25 grains from each plant were milled and assayed. The MGPA in milled flour was determined with a minimum of 4 biological replications.

### Statistics

The Welch’s *t*-test for unequal variance was employed to test significant differences in MGPA of all comparisons performed in this study.

## Results

### Field Trial Experiments

**FIGURE 2:**
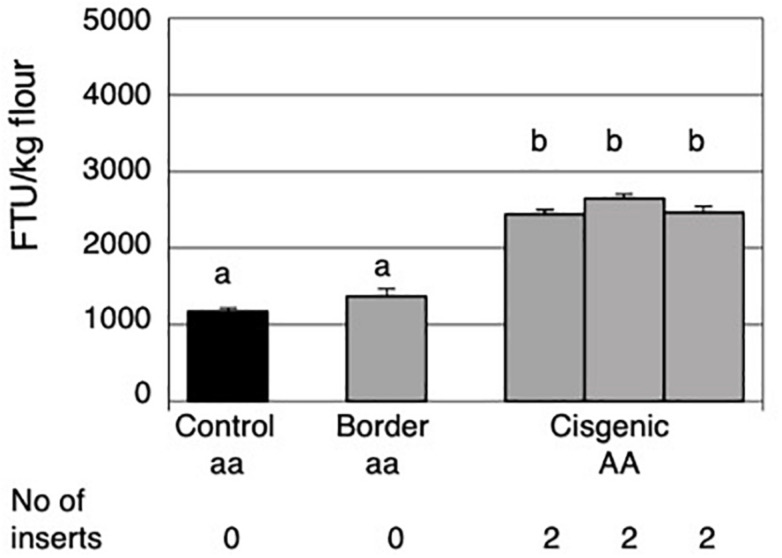
Mature grain phytase activities of grains from the control field plot, the border around the cisgenic field plot and the three areas within the cisgenic field plot. The control plot and the border contained non-transformed Golden Promise and the three areas with cisgenic plot contained cisgenic Golden Promise plants homozygous for a single *HvPAPhy_a* insert (PAP07). The error bars represent standard errors. The significance levels are shown with letters and values with the same letter(s) are not significantly different. The *HvPAPhy_a* inserts from PAP07 are referred to as A.

Field trials were performed for with the cisgenic plant PAP07, homozygous for one single *HvPAPhy_a* insert located on chromosome 3HS. A control field plot with non-transformed Golden Promise was also included. The control field plot was placed 150 m away from the cisgenic field plot in order to comply with the regulatory requirement of at least 100 m distance between the cisgenic barley field plot and other spring barley fields. As previously mentioned, barley already contains an endogenous *HvPAPhy_a* gene located on chromosome 5H. The average MGPA of Golden Promise seeds from the control field was 1170 FTU/kg flour. This was not significantly different from the average MGPA of the non-cisgenic border plants with 1374 FTU/kg flour (Figure 2).

The average MGPA in the three separate areas with cisgenic plants was 2434, 2643, and 2458 FTU/kg flour. Thus, the phytase activity seemed to be stable with no significant differences between the three areas. Overall there was an average increase of 2.2-fold in MGPA of the cisgenic field as compared to the MGPA of the non-cisgenic control field (Figure 2). This increase in MGPA was highly significant.

### Crosses for Stacking Experiments and MGPA of Parents and Progenies

#### Crosses and Genotyping

The stacking of the cisgenic inserts were performed by crossing the three different transformants (PAP07, PAP05, and PAP03) homozygous for the three different cisgenic inserts. The *HvPAPhy_a* insert in PAP07 is located on the short arm of chromosome 3H, the insert in PAP05 is located on the long arm of chromosome 3H and that the insert in PAP03 is located on the long arm of chromosome 2H (Supplementary Figure S1). The *HvPAPhy_a* inserts are referred to as A from PAP07, B from PAP05, and C from PAP03.

**FIGURE 3:**
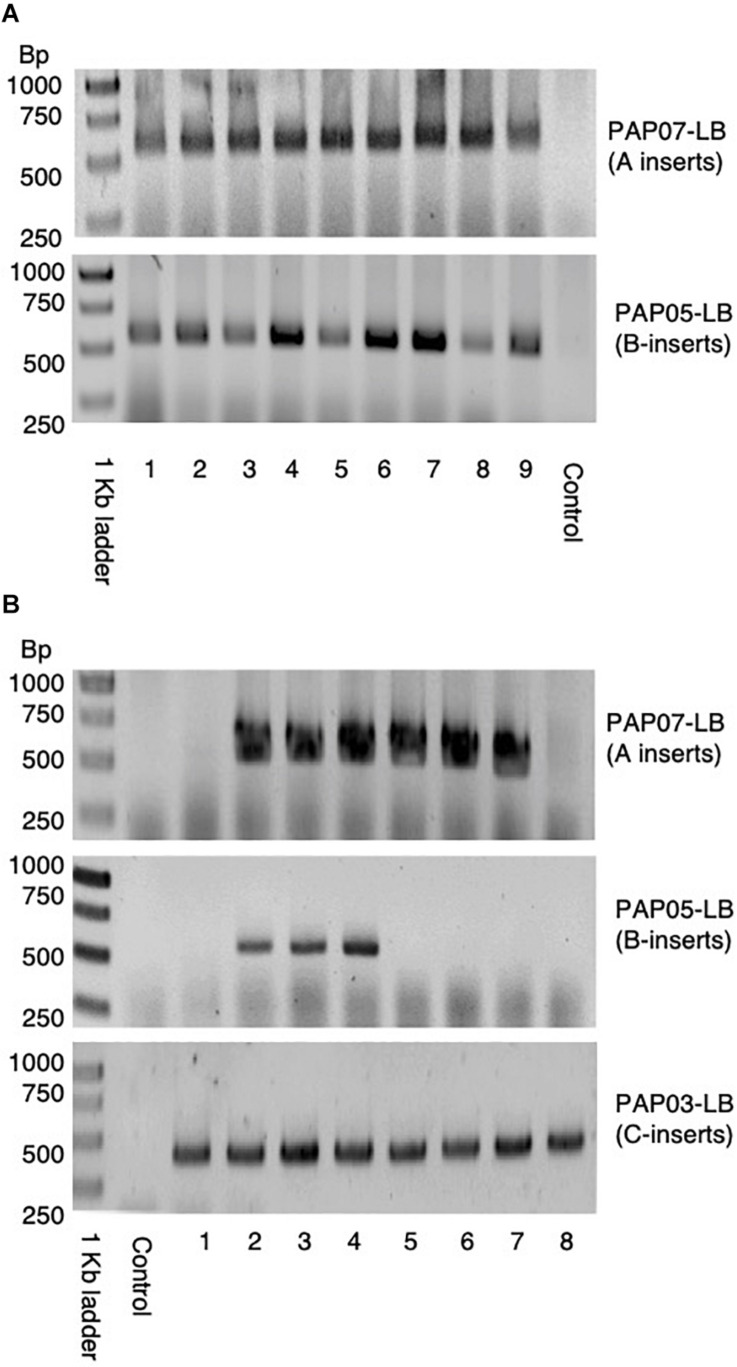
PCR of LB-flanking sequences of the progenies from the first and second cross used to stack the *HvPAPhy_a* inserts. **(A)** PCR of LB-flanking sequence of PAP07 and PAP05 in nine F_1_-progenies of the first cross between PAP07 and PAP05. **(B)** PCR of the LB-flanking sequence of PAP07, PAP05, and PAP03 in eight progenies of the second cross between F_1_-progenies (PAP07 × PAP05) and PAP03.

In the first cross between PAP07 and PAP05, nine progenies were investigated by PCR and they all showed the PCR amplification product of both *HvPAPhy_a* inserts (Figure 3A). In the second cross between F_1_ (PAP07xPAP05) and PAP03 eight progenies were recovered. Here, three plants had insert A, B and C, three had A and C and two had C inserts (Figure 3B).

#### Mature Grain Phytase Activities of the Parental Lines and Crosses

**FIGURE 4:**
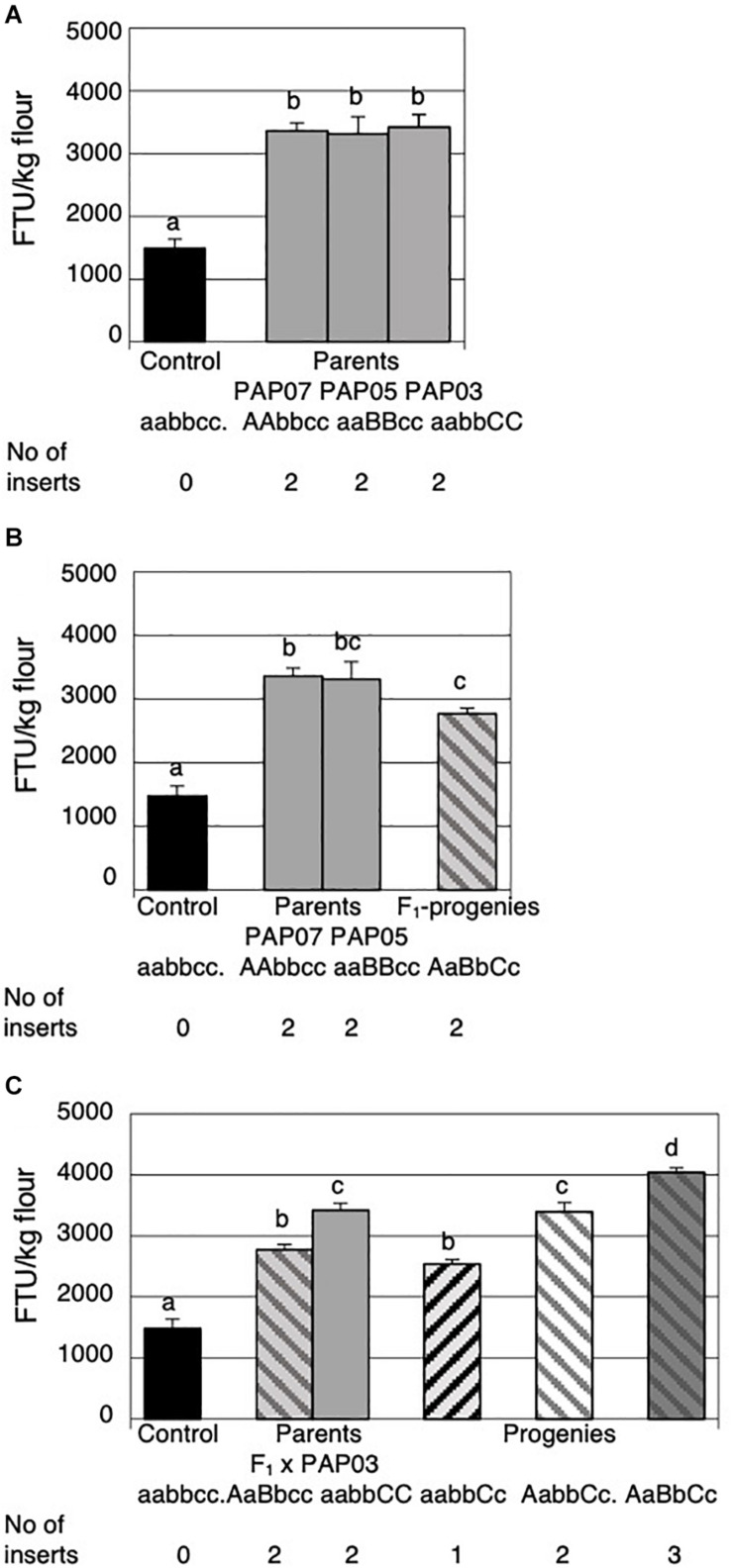
Mature grain phytase activities (MGPA) of grains from the parental lines and the progenies of the first and the second cross. **(A)** MGPA of the non-transformed control and the three parental lines PAP07, PAP05, and PAP03 each homozygous for a single *HvPAPhy_a* insert. **(B)** MGPA of the non-transformed control, the two parental lines PAP07 and PAP05 and the average of four F_1_-progenies obtained from the first cross. **(C)** MGPA of the non-transformed control, the two parental lines F_1_ (PAP07 × PAP05) and PAP03 and the average MGPA of the progenies obtained from the second cross. The error bars represent standard errors. The significance levels are shown with letters and values with the same letter(s) are not significantly different. The number of *HvPAPhy_a* inserts are indicated and referred to as A, B, and C for PAP07, PAP05, and PAP03, respectively.

The three parental plant lines and their progenies were grown in the greenhouse under environmental growth condition different from the plants grown in the field trial. The average MGPA of the non-transformed control and PAP07 is therefore 22% and 25% higher, respectively, than observed in the field (Figure 4A). Overall there was no significant difference in phytase activity between the three parental lines.

Four of the progenies from the initial cross were grown to maturity and the F_2_-grains were analyzed for MGPA (Figure 4B). The average MGPA of the progenies was compared to the MGPA of the parents homozygous for an extra insert of the *HvPAPhy_a* and the non-transformed Golden Promise containing one endogenous homozygous copy of the gene (Figure 4B). Both the parents and the F_1_-hybrid each with two extra copies of the *HvPAPhy_a* gene showed significantly higher MGPA than the non-transformed Golden Promise. The average MGPA of the F_1_-hybrids was not significantly different from the PAP05 parent but significantly lower than the PAP07 parent.

All eight progenies of the second cross were grown to maturity and analyzed for MGPA. The average MGPA for each genotype was compared with the MGPA of the non-transformed Golden Promise and the parents (Figure 4C). Again, non-transformed Golden Promise showed significantly lower MGPA’s than the parents and the progenies. The F_1_-hybrid parent with two *HvPAPhy_a* inserts (A and B) and the progenies containing only one extra *HvPAPhy_a* insert C did not show significantly difference in MGPA. The MGPA of the F_1_-hybrid parent were, however, significantly lower than the MGPA of the progenies containing two extra inserts (A and C) and also significantly lower than progenies containing all three *HvPAPhy_a* inserts A, B and C. The MGPA of the PAP03 parental line homozygous for a single *HvPAPhy_a* insert (C) was significantly higher than the MGPA of the progenies heterozygous for the C-insert. Also, the MGPA of the PAP03 parental line showed no significantly difference with progenies heterozygous for two extra *HvPAPhy_a* inserts (A and C). However, the increase in MGPA between the parental PAP03 line and the progenies containing all three *HvPAPhy_a* inserts was significant (from 3420 FTU/kg to 4041 FTU/kg). Overall, the results of the two crosses needed to generate a plant heterozygous for all three *HvPAPhy_a* inserts indicated an additive effect of the number of *HvPAPhy_a* inserts and the MGPA. However, an inconsistency was observed with the F_1_-hybrid parent containing two *HvPAPhy_a* inserts showing significantly lower MPGA than the PAP07 and PAP03 parents and progenies also containing two *HvPAPhy_a* inserts (Figures 4B,C).

### Genotypes of the Doubled Haploid (DH) Lines Obtained and MGPA of the DH-Lines

#### Genotyping

**FIGURE 5:**
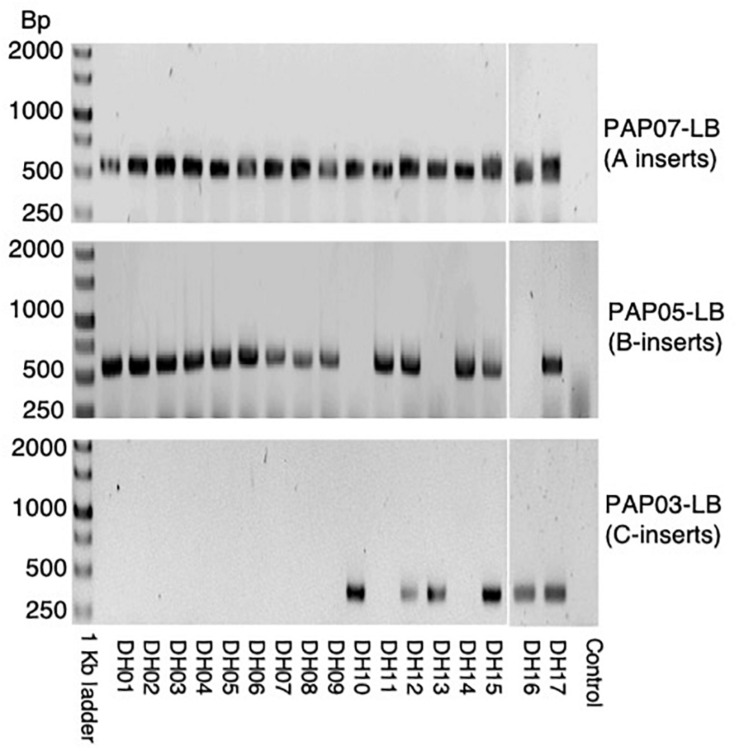
PCR of LB flanking sequences of PAP07, PAP05, and PAP03 in the 17 DH-plants produced from donor plants genotyped as A-B-C-.

Doubled haploid lines (DH-lines) were produced from F_2_-progenies of one of the F_1_-plants showing heterozygous inserts for A, B, and C (Figure 3B). Only F_2_-progenies showing all three *HvPAPhy_a* inserts by PCR of the LB-flanking sequences were used as donor plants (Supplementary Table S2). We obtained 17 DH-lines setting seeds signifying that the lines were derived from haploid microspores spontaneous doubled during anther culture. Investigation of these lines by PCR of the LB-flanking regions showed that eleven of the DH-lines were homozygous for the inserts A and B three of the DH-lines were homozygous for the inserts A and C and three of the DH-lines were homozygous for all three inserts from A, B, and C (Figure 5).

#### Mature Grain Phytase Activities of the DH-Lines

**FIGURE 6:**
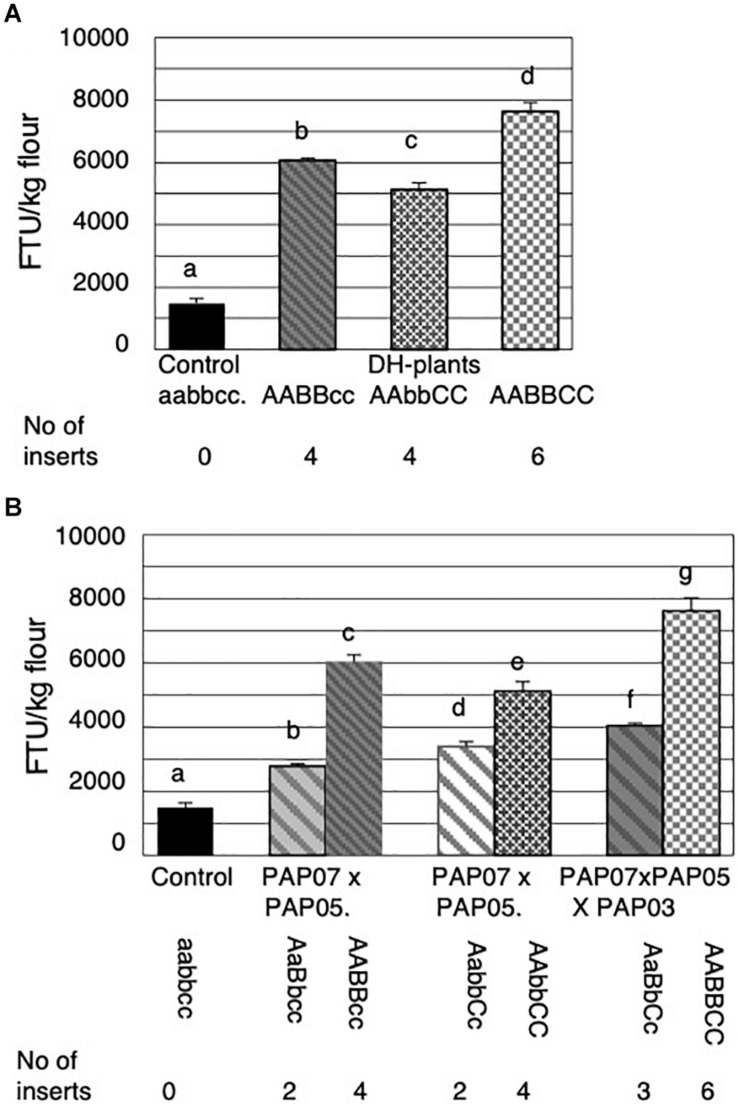
Mature grain phytase activities (MGPA) of grains from the DH-plants obtained through anther culture and comparisons of their corresponding heterozygous genotypes obtained in the second cross. **(A)** MGPA of the non-transformed control and DH plants obtained. **(B)** Comparison of homozygous and corresponding heterozygous genotypes. The error bars represent standard errors. The significance levels are shown with letters and values with the same letter(s) are not significantly different. The number of *HvPAPhy_a* inserts are indicated and referred to as A, B, and C for PAP07, PAP05, and PAP03, respectively.

The seeds of the DH-lines obtained were analyzed for MGPA. Plants with two stacked homozygous inserts showed further increases in MGPA compared to a single homozygous insert. However, there appeared to be a diverse combination effect between the lines with two inserts. The seeds of the DH-lines homozygous for *HvPAPhy_a* inserts A and B showed significantly higher MGPA than the seeds of the DH-lines homozygous for inserts A and C (Figure 6A). Still, seeds of the DH-lines homozygous for all three HvPAPhy inserts (A, B and C) showed significant higher MGPA than the two groups of DH-seeds homozygous for two *HvPAPhy_a* inserts A and B and A and C, respectively (Figure 6A).

An additive effect of the number of *HvPAPhy_a* inserts and MGPA was also clearly observed when comparing the MGPA of the DH-lines with the MGPA of their corresponding heterozygous plants (Figure 6B). Here the DH-lines all showed significantly higher MGPA than their corresponding heterozygous plants (Figure 6B). The average MGPA of the DH-lines with homozygous inserts A and B and of the DH-lines with homozygous inserts A and C was 6040 and 5132 FTU/kg flour, respectively. This corresponded to increases in MGPA of 2.2 and 1.5 fold, respectively, from the grains of the plants heterozygous for the corresponding *HvPAPhy_a* inserts. The average MGPA of the DH-lines with three homozygous inserts A, B and C was 7636 FTU/kg flour. This corresponded to an increase of 1.9-fold from the grains of the plants heterozygous for all three *HvPAPhy_a* inserts.

### Compiling Results

**FIGURE 7:**
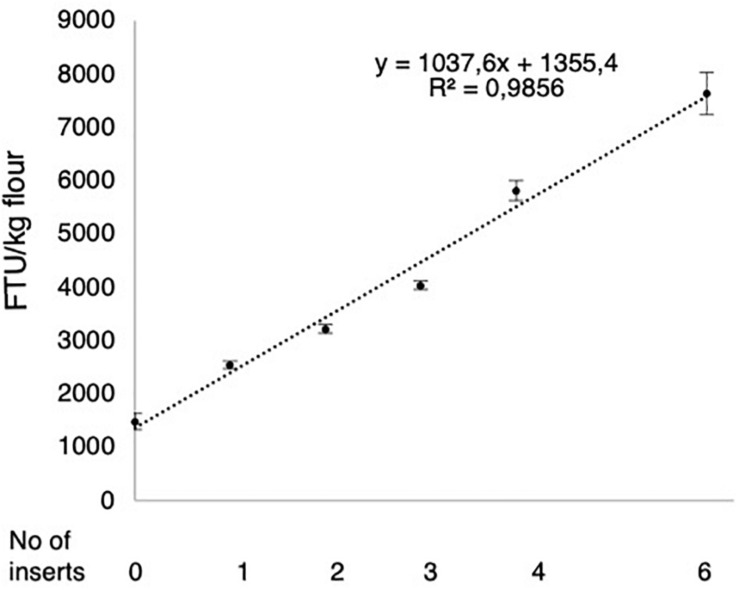
Correlation between the number of *HvPAPhy_a* inserts and the mature grain phytase activities. The error bars represent standard errors.

The MGPA values of all combinations of inserts included in this study were compiled and expressed as a function of the *HvPAPhy_a* insert number. The resulting correlation graph showed an estimated linear tendency line with the equation y = 1037.6 x + 1355.4 and a correlation value of 0.9856 (Figure 7). The equation estimated an average increase of 1037.6 and 2075.2 FTU/kg flour per heterozygous and homozygous *HvPAPhy_a* insert, respectively.

## Discussion

In the current study we have explored the potential of cisgenic barley with increased MGPA. First, we examined if the increase observed for greenhouse grown cisgenic barley is realized under field conditions. The grains of the PAP07 barley plants from the cisgenic field plot had a significant higher MGPA (from 2443 to 2643 FTU/kg) than the non-transformed field plot (from 1030 to 1252 FTU/kg). The variation in MGPA of cisgenic plants grown in the three separate areas was not significant. As an average, the increase in MGPA of the cisgenic field was 2.2 fold or 1342 FTU/kg higher than in the control field. The greenhouse data in the present study showed that the MGPA of the non-transformed control and plants of the cisgenic plant line PAP07 were 1484 and 3363 FTU/kg flour, respectively. This indicates that the MGPA in general is lower for barley plants grown in the field. The difference can most likely be attributed to different growth conditions between normal agricultural and greenhouse conditions with artificial watering, high fertilization level and controlled light and temperature regimes.

The potential of further MGPA increase was studied by horizontal stacking of *PAPhy_a* in barley. *HvPAPhy_a* originates from an ancient gene duplication shared by the Triticeae tribe cereals and time has allowed the evolution of different expression patterns of the paralogs *PAPhy_a* and *b*. More recent, duplications are found in *Secale* (including cultivated rye with 2 *PAPhy_a* type plus 1 *PAPhy_b* type) and in the polyploid *Triticum aestivum* (homeologs, 3 *PAPhy_*a type plus 3 *PAPhy_b* type). These duplicated genes are still highly conserved and has maintained their function (Madsen et al., 2013). Like barley, rye is a diploid. However, the two *PAPhy_a* loci in rye gives a MGPA of 2800 to 5800 FTU/kg flour as compared to barley with 700 to 1700 FTU/kg flour (Viveros et al., 2000; Madsen et al., 2013). In hexaploidy wheat, the loss of one of three *PAPhy_a* homeologs was associated with approximately 1/3 reduction in MGPA (Madsen et al., 2013).

The present study shows how multiplication of *PAPhy_a* confers an additive effect of the gene dose on the MGPA as also seems to be the case in rye and wheat which have natural *PAPhy_a* multiplications. Throughout the process of generating stacked *HvPAPhy_a* inserts in barley, the MGPA was measured in all the progenies from the crosses and the DH-lines containing different inserts and number of inserts. Although these measurements showed some variation, the combined data showed that there is a strong linear correlation between the number of *HvPAPhy_a* inserts and MGPA. The correlation equation showed that one extra homozygous insert in Golden Promise will increase the MGPA by 2075 FTU/kg flour. The final DH-plants with three homozygous inserts showed an actual MGPA of 7636 FTU/kg flour whereas the equation predicts a MGPA of 7581 FTU/kg flour. Moreover, there is no indication that 7636 FTU/kg is the limit. More inserts than the three homozygous inserts present in the current plants may accelerate MGPA even further and future studies will have to uncover how far stacking of *PAPhy_a* genes in barley can increase MGPA. In an earlier conventional transgenic study in barley where a *HvPAPhy_a* cDNA clone was expressed under the control of a constitutive 35S-promoter, the MGPA levels reached up to 29000 FT/kg flour (Holme et al., 2017a). However, a key difference between the current cisgene study and the 35S transgene study is the spatial expression pattern of the two promoters. The *PAPhy_a* promoter expression is almost exclusively confined to the aleurone and scutellum layers, whereas the 35S promoter is expressed in a constitutive way in the developing barley and thus also expressed in the endosperm of the seeds. Also, in the transgene study we could not distinguish between plants heterozygous and homozygous for the inserts and only a few plants were investigated for the number of *HvPAPhy_a* inserts. However, when comparing the increase in MGPA per 35S-controlled *HvPAPhy_a* insert with the increase per cisgenic *HvPAPhy_a* insert, at least 3 times higher increases in MGPA was obtained when using the 35S-promoter. Still, there might be more phytase present in the aleurone layer in the cisgenic plants due to the aleurone tissue specific promoter in the *HvPAPhy_a* gene and there might also be a major benefit of the phytase location in the aleurone layer being close to the phytate (Brejnholt et al., 2011).

In the present study, the potentials of cisgene gene stacking has been demonstrated. We used immature embryos from the barley variety Golden Promise as transformation target as this is the only barley cultivar that gives a reasonable yield of transformants with immature embryos (Harwood, 2012). Introgression of the cisgenic *HvPAPhy_a* inserts to elite barley varieties is a prerequisite for a general use of the extra *HvPAPhy_a* inserts. However, the *HvPAPhy_a* inserts A, B and C are unlinked and segregates independently. Still, as we now know that the three *HvPAPhy_a* inserts will increase the MGPA to a high level, it might be preferable to transform Golden Promise with three cisgenic *HvPAPhy_a* inserts combined within one T-DNA. By stacking the cisgenic *HvPAPhy_a* inserts in a single T-DNA, only one marker is needed in the introgression process to other varieties. The number of offspring plants to screen would then be reduced and the linkage drag would be as small as possible. In potato, transformation with a single *Agrobacterium* vector containing three different cisgenic late blight resistance genes with the same combined size as the combined size of three cisgenic *HvPAPhy_a* inserts were successfully used to achieve cisgenic potatoes with durable resistance to late blight (Haverkort et al., 2016). Genes stacked in this way would be similar to naturally occurring tandem repeat duplications which result from unequal crossing over (Zhang, 2003). This approach could be extended to the simultaneous use of site directed nucleases (e.g., CRISPR/Cas9) to insert the combined three *HvPAPhy_a* inserts at a favorable place in the genome (Yin et al., 2017). Also, change of immature embryos to transformation targets less genotype dependent, like ovules or microspores, might exclude the transfer of the cisgenes to elite cultivars via time consuming backcrosses (Kumlehn et al., 2006; Holme et al., 2008).

The results of the current study demonstrate that MGPA in barley can be increased significantly by cisgene stacking of the *PAPhy_a* genes. According to a scientific opinion published by EFSA (European Food Safety Authority), an extra supplement of 500 to 1000 FTU/kg feed of microbial phytase is recommended in pig feed when the diet is based on wheat, barly and soybean (EFSA, 2010). However, the efficacy of endogenous cereal phytase is significantly reduced if the grain is processed to pelleted feed (Slominski et al., 2007) and a higher increase in MGPA would therefore be required for such an application. Furthermore, feed consisting of 100% barley is rare and increased MGPA would enable farmers to only partially replace conventional barley with cisgenic phytase barley. For HighPhy wheat with an MGPA of around 6200 FTU/kg flour it was found that by replacing 1/3 standard field grown wheat (with a MGPA of 1060 FTU/kg flour) with HighPhy in broiler feed an even better improvement of Ca and P digestibility was achieved than if the feed was supplemented with the standard dose of microbial phytase (Scholey et al., 2017). The current cisgene stacking reached phytase levels higher than the 6200 FTU/kg for the HighPhy wheat and constitutes a tangible improvement of MGPA in barley by non-transgenic means.

We have uncovered the potential of cisgene phytase barley as a field crop. Open field experiments confirmed a stable increased level of MGPA. *HvPAPhy_a* cisgenes were stacked by crossing and double haploid production from progenies. Plants with 1, 2, 3, 4, and 6 *HvPAPhy_a* inserts were generated and displayed a clear linear increase of MGPA. Our results provide insight into the potential of stacking of cisgenes in crops and suggests that future efforts could be directed toward understanding whether cisgene stacking of valuable traits is a versatile strategy for crop improvement.

## Data Availability Statement

The raw data supporting the conclusions of this article will be made available by the authors, without undue reservation.

## Author Contributions

IH and HB-P designed the project. IH, CM, and TW conducted the experiments. IH analyzed the data. IH, HB-P, and CM wrote the initial manuscript which was carefully revised by TW. All authors read and approved the final manuscript.

## Conflict of Interest

The authors declare that the research was conducted in the absence of any commercial or financial relationships that could be construed as a potential conflict of interest.
