# Supplementary material for: Horizontal Stacking of PAPhy_a Cisgenes in Barley Is a Potent Strategy for Increasing Mature Grain Phytase Activity
Source: Front Plant Sci. 2020 Oct 23;11:592139. doi: 10.3389/fpls.2020.592139 (PMC7644513; doi:10.3389/fpls.2020.592139)
Supplement: Supplementary file 1 [file Table_1.DOCX]

**Figure S1.** Field plot design. Control field plot with non-transformed Golden Promise (**A**) and the cisgenic field plot (**B**). Total area per plot is 6 x18 m (108 m^2^). Plots were surrounded by a border of 1.5 m non-transformed Golden Promise barley (Grey) for pollen catching, leaving the actual experimental plots to 3 x 15 m = 45 m^2^. The cisgenic plot was divided into three areas, which were harvested separately.

**A**

CAGTGCCAGTATCCAGTGAGGCTGGCCGGCCGTGTCCCGCACTGTCGTCGCCGCCGCCGGCAGAACAAATCGCTAGGAAGGAAGGAACACCGGCTGGCGAATGGGGCTGGAGGGGGCGAATTTGGGGGGAGAAATCTGCGAGGAGACAAGGCGACGGGAAGGATGAACGTTGGAAACGGGGCAAATCCACGATTTTATTAGTCGGTTTATATAGGAGACACCAGCACTAATGGCTGTTCTACCGAATTGGCCGGGAGAAGGCCCCGACGTCTCCCGTCACACACAGTCTGCTGAAAACCCTCACCTTTGAGTAGATTTTAGATTTAGGGGTGTCTGACTCTTGTCTAGATAAGAAATACTGTAATTATGTCTTGTGTAACTCCTACACCATTGAATTTTACGTGGAGATTCATAGGATCTTTTTTCTTTTGCCTTTTGTTTGGTTAGAATATTACAGTATATAGGTTAGATGAGACTTTGTTAATTGTCATTTACTGCCTTCGTTCGTAGATGTAAGTTTTTTTTTAAAATTACACTATTAAACAACATACGGATGTACATAGATATATTTTAAAATATAAATTTATTTATTTTATTTTGTA*TGCAGCCTCCTGTTAAAATGTCCAA*AAAGGTTTATACTATTTAGGAAGTGAGAGAGTAGATGGGAATTAACAACTTCATTGAATTTAACCTTGAAATGCAATACGTACTGTTTAGTGTGTGTATGGCCGATAGATA

*atattgtggtgtaaac*gttcctgcggccgc

atcttgggcaacatatcaggggcagcgccattgccctgcgactgacggcggcggtggaggagcttggggcagacatgagctgagaacgacgagagagaggagtggtggcgggcgagacagaggagcgacatgattgaagaagagcagcgggattgaggattagggattcctgcgattttacacttgacctctccataaaagattggcctaatcgaagctgagaacgtggaggtcaacaagtggtcaaacgagcctgtacgcaccgcatacgagcaacagtgatcggattttcacgtcacatcgtatatagtgatcgtaaaagccatattctaaagttggatgaccgtattgtgcttccat*gtcaactgcaaggaccgtgagtgt*atttatctctaaaat

**B**

AGTGGTAGATAGGACTCGAGAAAATATTAGTCTTACCTTTAGCTCCTTATGGGTTCGACACTTTATACTTATCACTTCCATCTTCGGAAAGTTCTACGATGATTCCTTGCACTTGGGAATTATCAAAGGGTCGTCGAGGCCTGTCACGCGGAGGAAATGGTGGCGACAGAGGCATCTGATGCGGCGGCGCGGGCGACCGGAGAGAAGGAAGCCATTCGCGTCCGTATTTTGAAGAAACGTATTTTGAAGAAACAGCAACGGAGGAACACGCGCGGCCTCGCCCGAGATCAGAA*TCGTGCGGTCCGCGAAATGG*CTGGATTGCCACAAAAGGAGGAGGAGGAGGTGAGCGACGATGAGAACAGCTCCGATGACAAGAAGATCCGGTTGGATCCATATTAGGTCTTGGACTGGTACTTAGGCGAGAAAGACAGCAAGGTCTCCGGGAAGGACAAGGAGAGTCGTGGCTAATGTCCACCGTAGTCATACATGTTAAATTTTGATAGTCCAATGAGATGTTAATATTAATGGAGTAGCCGGATGATATAT

*atatattgtggtgtaaac*gttcctgcggccgc

atcttgggcaacatatcaggggcagcgccattgccctgcgactgacggcggcggtggaggagcttggggcagacatgagctgagaacgacgagagagaggagtggtggcgggcgagacagaggagcgacatgattgaagaagagcagcgggattgaggattagggattcctgcgattttacacttgacctctccataaaagattggcctaatcgaagctgagaacgtggaggtcaacaagtggtca*aacgagcctgtacgcaccgc*atacgagcaacagtgatcggattttcacgtcacatcgtatatagtgatcgtaaaagccatattctaaagttggatgaccgtat

**C**

GATCATATATTTTATATTACTAGATAAATGAGCATTCTTTGCAATGGGGTACAAATATTTTGTACACAATTGATACTTCTCCGACATATCTATATATCTTTTAATATTTCATGCTATTATCATACCACTTCACATATTTTTTAGCAACAATTTATGTTATTTTTATTTGGATTAACATATT*TGATAGAGGCGAAGGTGTCC*CGATATTTCAATGAGATGACAACTATCGATTTGGTGGAGACGAATTTGACGTTCCGACTACAAACGTGCACGACGTTGCGCCTTAGCAATCGCTAAACCAACTCCAAGAGGTTATGGACCACGCCAGAGCACGATCAACCTGACCACGAAAGTCTATTCCTGCAAGCAATCGAAGAACAAGTAAGAATATGATATTGCAATCTGAATATTGTGAATATAGATAAAGTATTGATAAGGGTGGGGATCCGAAAGCGGTCTTAGTCTGGTC

*gtaaac*gttcctgcggccgc

atcttgggcaacatatcaggggcagcgccattgccctgcgactgacggcggcggtggag*gagcttggggcagacatgag*ctgagaacgacgagagagaggagtggtggcgggcgagacagaggagcgacatgatt

**Figure S2.** Left-border flanking sequences of the three *HvPAPhy_a* inserts.

The whole LB-flanking sequence of each insert showed 99 % homology to a corresponding sequence in Barke using the Webblast ipk-gatersleben.de/barley and for each flanking area no other homologies were found for the whole sequences. The LB-flanking sequence identified for inserts in PAP07 (**A**), PAP 05 (**B**) and PAP03 (**C**) showed 99 % homology to a sequence on chromosome 3HS, 3HL and 2HL, respectively. Capital letters: LB-flanking sequences; LB-sequences integrated (grey), multiple cloning site (yellow). Lower case letters: Sequences of the first part of the *HvPAPhy_a* promoter. Primers used to amplify the flanking sequences in the current study are shown in red (Table S1).

**Table S1.** Sequences of forward and reverse primers, PCR-product size and conditions for the PCR reactions for the Left T-DNA border flanking areas of the *HvPAPhy_a* inserts in PAP07, PAP05 and PAP03.

| Primers | Sequences | PCR-products | Conditions for PCR reactions |
| --- | --- | --- | --- |
| Primers for the amplification of the Left border (LB) of PAP07 | Forward: 5’-TGCAGCCTCCTGTTAAAATGTCCAA-3’  Reverse: 5’-ACACTCACGGTCCTTGCAGTTGAC-3’ | 550 bp | An initial step of 95°C for 5 min, then 35 cycles of 95°C for 30 s, 60°C for 30s, 72°C for 30s and then 72°C for 7 min |
| Primers for the amplification of the LB of PAP05 | Forward: 5’-TCGTGCGGTCCGCGAAATGG-3’  Reverse: 5’-GCGGTGCGTACAGGCTCGTT-3’ | 551 bp | Do |
| Primers for the amplification of the LB of PAP03 | Forward: 5’- TGATAGAGGCGAAGGTGTCC-3’  Reverse: 5’- CTCATGTCTGCCCCAAGCTC-3’ | 387 bp | Do |

| Generations | Parents  or donor material for  DH-production | | Crosses or DH-production | | | Plants selected for further crossing /selfing or DH-production | Selected for MGPA analysis |
| --- | --- | --- | --- | --- | --- | --- | --- |
|  |  |  | Expected genotype of crosses | Identified genotypes by PCR | |  |  |
| First cross | PAP07 (AAbb) | PAP05 (aaBB) | All AaBb | Nine F1 seeds germinated  All AaBb | | Two randomly chosen F1-plants | Seeds from four F1-plants. |
| Second cross | Two F1-plants  (AaBb) | PAP03 (aabbCC) | 1: AaBbCc;  1: AabbCc;  1: aaBbCc;  1: aabbCc | Eight F1 seeds  3: AaBbCc;  3: AabbCc;  0: aaBbCc;  2: aabbCc | | One plant (AbBbCc) selfed and seeds used for DH-production | Seeds of all eight F1-plants |
| DH-production | Twenty five F2-seeds (from self-pollinated AaBbCc) germinated and genotyped for A-,B-,C- by PCR of flanking sequences. Seven plants containing all three inserts used for anther cultures. Spikes with anthers for DH-production were randomly chosen among these seven plants. | | If all combinations present in the seven plants then with random ratios:  AABBCC  AABBcc  AAbbCC  AAbbcc  aaBBCC  aaBBcc  aabbCC  aabbcc | Total green plants  10: AABBCC  14: AABBcc  5: AAbbCC  0: AAbbcc  0: aaBBCC  0: aaBBcc  0: aabbCC  0: aabbcc | Plants setting seeds  3: AABBCC  11: AABBcc  3: AAbbCC | 3: AABBCC  11: AABBcc  3: AAbbCC | Seeds of the 17 DH plants |

**Table S2.** Crosses and production of DH-lines used for the stacking of the three *HvPAPhy_a* inserts A-, B- and C- from the plants PAP07, PAP05 and PAP03, respectively.
